# Supplementary material for: In-silico analysis of myeloid cells across the animal kingdom reveals neutrophil evolution by colony-stimulating factors
Source: eLife. 2020 Nov 25;9:e60214. doi: 10.7554/eLife.60214 (PMC7717901; doi:10.7554/eLife.60214)
Supplement: Supplementary file 1. [file elife-60214-supp1.docx]

| Gene name | Protein name |
| --- | --- |
| CSF1R | Colony stimulating factor receptor 1/ Macrophage colony stimulating factor receptor |
| CSF1 | Colony stimulating factor receptor 1/ Macrophage colony stimulating factor |
| IL34 | Interleukin 34 |
| CSF3R | Colony stimulating factor receptor 3/ Granulocyte colony stimulating factor receptor |
|  | Colony stimulating factor receptor 3/ Granulocyte colony stimulating factor |
