# Supplementary material for: In-silico analysis of myeloid cells across the animal kingdom reveals neutrophil evolution by colony-stimulating factors
Source: eLife. 2020 Nov 25;9:e60214. doi: 10.7554/eLife.60214 (PMC7717901; doi:10.7554/eLife.60214)
Supplement: Supplementary file 2. [file elife-60214-supp2.docx]

| Family member | Developmental stage expression | Impact on neutrophil population in Cebpx^-/-^ model | Functional role in granulopoiesis | References |
| --- | --- | --- | --- | --- |
| *Cebpα* | HSCs, CMPs, GMPs | Loss of primary granules including; MPO and ELANE | *Cebpα* is required for transition from CMP to GMP. In its absence, granulopoiesis is blocked and there is an accumulation of CMPs. | [1-4] |
|  |  | Loss of specifc granules including lactoferrin |  |  |
|  |  | Loss of CSF3R and IL6R |  |  |
|  |  | Loss of granulocytes but monocytes were retained. |  |  |
|  |  | Newborn mice lack granulocytes but retain monocytes |  |  |
|  |  | BM from adult mice have markedly less GMPs |  |  |
| *Cebpβ* | GMPs - mature neutrophils | -/- mice had increased susceptibility to systemic Candida Albicans | *Cebpβ* is essential for emergency and/or cytokine induced granulopoiesis , however it is not required for steady-state haematopoiesis. | [5-8] |
|  |  | -/- mice also had increased susceptibility to listeria and salmonella infections. |  |  |
|  |  | There is no mobilization of granulocytes in response to systemic fungal infection or cytokine stimuli |  |  |
|  |  |  |  |  |
|  |  |  |  |  |
|  |  |  |  |  |
| *Cebpε* | Promyelocytes - mature neutrophils | Young -/- mice showed no difference in behaviour compared with wild-type and heterozygous littermates. | *Cebpe* is required for the terminal differentiation of granulocytes and it's deficiancy blocks granulocytic maturation but did not affect any of the other haemopoietic lineages. | [9-14] |
|  |  | -/- mice have a marked increase in granulocytic progenitors in BM. |  |  |
|  |  | Increased number of hyposegmented atypical neutrophils in the peripheral blood. |  |  |
|  |  | Neutrophil popultions from -/- mice sjowed no NADPH oxidase activity, reduced phagocytosis and impaired migratory function. |  |  |
|  |  | Neutrophil popultions from -/- mice lacked specific and tertiary granules including lactoferrin and gelatinase B. They also display delayed chemotaxis. |  |  |
|  |  | -/- mice suffer from severe or fatal repeat chronic bacterial infections and died within 3-5 months under specific pathogen-free conditions. |  |  |
| *Cebpβ Cebpε (double knockout model)* | GMP - mature neutrophils | Early development was found to be normal in double knockout (*bbee*) mice | There seems to be an additive effect when both *Cebpβ* and *Cebpε* are absent. There is a block at the myelocyte to metamyelocyte stage of differentiation. Both *Cebpβ* and *Cebpε* regulate the expression of cytokines, such as IL-8, in human neutrophils. | [15] |
|  |  | *bbee* haematopoietic progenitor cells had an impaired ability to form colonies. |  |  |
|  |  | *bbee* mature neutrophils lacked granules and presented with atypical bi-lobed nuclei. |  |  |
|  |  | *bbee* mice died between 2-3 months of age. They presented with splenomegaly and several different strains of systemic bacterial infection. |  |  |
|  |  |  |  |  |
|  |  |  |  |  |

1. Smith, L.T., et al., *PU.1 (Spi-1) and C/EBP alpha regulate the granulocyte colony-stimulating factor receptor promoter in myeloid cells.* Blood, 1996. **88**(4): p. 1234-47.

2. Ma, O., et al., *Granulopoiesis requires increased C/EBPα compared to monopoiesis, correlated with elevated Cebpa in immature G-CSF receptor versus M-CSF receptor expressing cells.* PLoS One, 2014. **9**(4): p. e95784.

3. Zhang, P., et al., *Enhancement of hematopoietic stem cell repopulating capacity and self-renewal in the absence of the transcription factor C/EBP alpha.* Immunity, 2004. **21**(6): p. 853-63.

4. Ford, A.M., et al., *Regulation of the myeloperoxidase enhancer binding proteins Pu1, C-EBP alpha, -beta, and -delta during granulocyte-lineage specification.* Proc Natl Acad Sci U S A, 1996. **93**(20): p. 10838-43.

5. Zhang, P., et al., *Induction of granulocytic differentiation by 2 pathways.* Blood, 2002. **99**(12): p. 4406-12.

6. Hirai, H., et al., *C/EBPbeta is required for 'emergency' granulopoiesis.* Nat Immunol, 2006. **7**(7): p. 732-9.

7. Screpanti, I., et al., *Lymphoproliferative disorder and imbalanced T-helper response in C/EBP beta-deficient mice.* EMBO J, 1995. **14**(9): p. 1932-41.

8. Tanaka, T., et al., *Targeted disruption of the NF-IL6 gene discloses its essential role in bacteria killing and tumor cytotoxicity by macrophages.* Cell, 1995. **80**(2): p. 353-61.

9. Yamanaka, R., et al., *Impaired granulopoiesis, myelodysplasia, and early lethality in CCAAT/enhancer binding protein epsilon-deficient mice.* Proc Natl Acad Sci U S A, 1997. **94**(24): p. 13187-92.

10. Morosetti, R., et al., *A novel, myeloid transcription factor, C/EBP epsilon, is upregulated during granulocytic, but not monocytic, differentiation.* Blood, 1997. **90**(7): p. 2591-600.

11. Verbeek, W., et al., *C/EBPepsilon -/- mice: increased rate of myeloid proliferation and apoptosis.* Leukemia, 2001. **15**(1): p. 103-11.

12. Chih, D.Y., et al., *Modulation of mRNA expression of a novel human myeloid-selective CCAAT/enhancer binding protein gene (C/EBP epsilon).* Blood, 1997. **90**(8): p. 2987-94.

13. Lekstrom-Himes, J. and K.G. Xanthopoulos, *CCAAT/enhancer binding protein epsilon is critical for effective neutrophil-mediated response to inflammatory challenge.* Blood, 1999. **93**(9): p. 3096-105.

14. Gombart, A.F., et al., *Aberrant expression of neutrophil and macrophage-related genes in a murine model for human neutrophil-specific granule deficiency.* J Leukoc Biol, 2005. **78**(5): p. 1153-65.

15. Akagi, T., et al., *In vivo deficiency of both C/EBPβ and C/EBPε results in highly defective myeloid differentiation and lack of cytokine response.* PLoS One, 2010. **5**(11): p. e15419.
