# Supplementary material for: In-silico analysis of myeloid cells across the animal kingdom reveals neutrophil evolution by colony-stimulating factors
Source: eLife. 2020 Nov 25;9:e60214. doi: 10.7554/eLife.60214 (PMC7717901; doi:10.7554/eLife.60214)
Supplement: Supplementary file 3. [file elife-60214-supp3.docx]

| Species Group | n | Refs |
| --- | --- | --- |
| Placental | 11 | [1-3] |
| Marsupial | 5 | [4-6] |
| Monotremata | 1 | [7] |
| Aves | 5 | [8, 9] |
| Crocodilia | 3 | [10, 11] |
| Testudines | 4 | [12, 13] |
| Squamata | 6 | [14, 15] |
| Anura | 2 | [16] |
| Jawed Fish | 3 | [17, 18] |

1. Martin, S., et al., *Hematological Survey of Common Neotropical Bat Species from Costa Rica.* Journal of Zoo and Wildlife Medicine, 2011. **42**(3): p. 382-391.

2. Nemzek, J.A., et al., *Differences in normal values for murine white blood cell counts and other hematological parameters based on sampling site.* Inflammation Research, 2001. **50**(10): p. 523-527.

3. Bain, B.J., *Ethnic and sex differences in the total and differential white cell count and platelet count.* Journal of Clinical Pathology, 1996. **49**(8): p. 664.

4. McKenzie, S., E.M. Deane, and L. Burnett, *Haematology and Serum Biochemistry of the Tammar Wallaby, Macropus eugenii.* Comparative Clinical Pathology, 2002. **11**(4): p. 229-237.

5. Viggers, K.L. and D.B. Lindenmayer, *Hematological and Plasma Biochemical Values of the Greater Glider in Australia.* Journal of Wildlife Diseases, 2001. **37**(2): p. 370-374.

6. Bennett, M.D., et al., *Hematologic characteristics of captive western barred bandicoots (Perameles bougainville) from Western Australia.* Veterinary Clinical Pathology, 2007. **36**(4): p. 348-353.

7. Whittington, R.J. and T.R. Grant, *Hematological Changes in the Platypus (Omithorhynchus anatinus) Following Capture.* Journal of Wildlife Diseases, 1995. **31**(3): p. 386-390.

8. Polo, F.J., et al., *Hematological Values for Four Species of Birds of Prey.* The Condor, 1992. **94**(4): p. 1007-1013.

9. Hawkey, C., et al., *Haematological changes in domestic fowl (Gallus gallus) and cranes (Gruiformes) with Mycobacterium avium infection.* Avian Pathology, 1990. **19**(2): p. 223-234.

10. Lovelya, C.J., J.M. Pittmanb, and A.J. Lesliec. *Artikel Normal haematology and blood biochemistry of wild Nile crocodiles ( Crocodylus niloticus ) in the Okavango Delta , Botswana*. 2007.

11. Sergio, E.P., W. Manuel, and R.J. Elliott, *HEMATOLOGIC AND PLASMA BIOCHEMICAL REFERENCE INTERVALS FOR MORELET'S CROCODILES (<span class="genus-species">CROCODYLUS MORELETII</span> ) IN THE NORTHERN WETLANDS OF CAMPECHE, MEXICO.* Journal of Wildlife Diseases, 2011. **47**(3): p. 511-522.

12. Bielli, M., et al., *Hematological values for adult eastern Hermann's tortoise (Testudo hermanni boettgeri) in semi-natural conditions.* J Vet Diagn Invest, 2015. **27**(1): p. 68-73.

13. Lewbart, G.A., et al., *Blood gases, biochemistry, and hematology of Galapagos green turtles (Chelonia mydas).* PLoS One, 2014. **9**(5): p. e96487.

14. Lisičić, D., et al., *Biochemical and hematological profiles of a wild population of the nose-horned viper Vipera ammodytes (Serpentes: Viperidae) during autumn, with a morphological assessment of blood cells.* Zoological Studies, 2013. **52**(1): p. 11.

15. Moller, C.A., T. Gaál, and J.N. Mills, *The hematology of captive Bobtail lizards (Tiliqua rugosa): blood counts, light microscopy, cytochemistry, and ultrastructure.* Veterinary Clinical Pathology, 2016. **45**(4): p. 634-647.

16. Forzán, M.J., et al., *Hematologic reference intervals for Rana sylvatica (Lithobates sylvaticus) and effect of infection with Frog Virus 3 (Ranavirus sp., Iridoviridae).* Vet Clin Pathol, 2016. **45**(3): p. 430-43.

17. de Oliveira, A.T., et al., *Hematological parameters of three freshwater stingray species (Chondrichthyes: Potamotrygonidae) in the middle Rio Negro, Amazonas state.* Biochemical Systematics and Ecology, 2016. **69**: p. 33-40.

18. Dove, A.D.M., J. Arnold, and T.M. Clauss, *Blood cells and serum chemistry in the world’s largest fish: the whale shark Rhincodon typus.* Aquatic Biology, 2010. **9**(2): p. 177-183.
