# Supplementary material for: In-silico analysis of myeloid cells across the animal kingdom reveals neutrophil evolution by colony-stimulating factors
Source: eLife. 2020 Nov 25;9:e60214. doi: 10.7554/eLife.60214 (PMC7717901; doi:10.7554/eLife.60214)
Supplement: Supplementary file 4. [file elife-60214-supp4.docx]

|  | Gene id/ Accesscion number | | | | | | | |
| --- | --- | --- | --- | --- | --- | --- | --- | --- |
|  | CEBPA | CEBPB | CEBPE | CSF1 | CSF1R | IL34 | CSF3 | CSF3R |
| Coelacanth | 102355541 | 102358307 | 102353844 | n/a | 102349790 | 102348256 | ENSLACG00000014917 | 102353589 |
| Australian ghost shark | 103188072 | 103172865 | n/a | n/a | n/a | n/a | n/a | n/a |
| Whale shark | n/a | 109916047 | n/a | n/a | n/a | 109911203 | n/a | n/a |
| Lamprey | n/a | 116945973 | n/a | n/a | 116955069 | n/a | n/a | n/a |
| Hagfish | ENSEBUG00000002399 | n/a | n/a | n/a | ENSEBUG00000015406 | n/a | n/a | n/a |
| Tropical clawed frog | 496454 | 619595 | 100038238 | 100492777 | 493543 | 101734802 | 100497396 | 100498116 |
| African clawed frog | 379967 | 734434 | 108714863 | 101243553 | 101243554 | 108715469 | 108700769 | 446770 |
| High Himalaya frog | 108801521 | 108798393 | 108797955 | 101243553 | 108785796 | 108802276 | 108702485 | 108804707 |
| Two-lined Caecilians | 115095103 | 115098040 | 115078924 | 115073984 | 115079743 | 115095363 | 115073650 | 115073078 |
| Microcaecilia Unicolor | 115470849 | 1155475677 | 115457776 | 115481878 | 115476576 | 115470426 | 115480063 | 115481384 |
| Three toed box turtle | 112120337 | 112111696 | 112122068 | 112115202 | 112106501 | 112115025 | 112110288 | 112108687 |
| Painted turtle | 101951046 | 101932390 | 101944182 | 101951664 | 101940831 | 101939784 | 101939784 | 101942477 |
| Green Sea turtle | 102945949 | 102946774 | 102930218 | 102929703 | 102937993 | 102937757 | 102929617 | 102931914 |
| Chinese Softshelled turtle | 102448303 | 106732445 | n/a | 102451139 | 102460494 | 102447970 | 102459061 | 102460143 |
| Burmese python | n/a | 103064423 | 112542412 | 103049576 | 103051818 | n/a | 103067892 | 112542138 |
| Mainland tiger snake | 113420299 | 113410417 | 113422278 | n/a | 113423624 | n/a | 113419986 | 113416023 |
| Eastern Brown snake | 113445329 | 113434361 | 113448737 | 113440646 | 113433809 | n/a | 113447404 | 113437695 |
| Central bearded dragon | 110081279 | 110071801 | 110087832 | 110087976 | 110086091 | n/a | 110085575 | 110082761 |
| Gecko | 107121021 | 107105657 | 107106288 | 107106414 | 107116323 | n/a | 107120752 | 107110150 |
| Anole | 100562398 | 100560183 | 100554198 | 103278674 | 100555979 | n/a | 103279240 | 103281080 |
| Gharial | 109289287 | n/a | n/a | 109295181 | 109289895 | n/a | 109290478 | 109294237 |
| Australian saltwater crocodile | n/a | 109318702 | n/a | 109317538 | 109310160 | 109310451 | 109320297 | 109318569 |
| American Alligator | 102575011 | 102565052 | 102560455 | 102560814 | 102565932 | 102571592 | 102564831 | 102560832 |
| Chinese Alligator | n/a | n/a | 102381769 | 102376334 | 102373216 | 102368968 | 102380087 | 102369421 |
| Emu | 112987621 | 112982872 | n/a | 112990372 | 112996913 | 112987442 | 112993873 | 112985221 |
| Ostrich | n/a | n/a | n/a | n/a | 104142216 | 104147241 | n/a | 104149428 |
| Kiwi | 112963156 | 112961791 | n/a | 112961471 | 112964855 | 112976880 | 112969542 | 112975657 |
| Mallard | 110351216 | 106019185 | n/a | 101804317 | 101802224 | 113844894 | n/a | 101791747 |
| Chicken | 427549 | 396185 | n/a | 100499189 | 396406 | 100858424 | 396216 | 419620 |
| Helmeted Guinea Fowl | 110404144 | 110408158 | n/a | 110388070 | 110405187 | 110404544 | 110388696 | 110387487 |
| Turkey | n/a | n/a | n/a | 100549089 | 100549842 | 100546208 | 100545706 | 100542145 |
| Platypus | 100078506 | 103165264 | 100087091 | 100073393 | 100079755 | 100075178 | 114815000 | 100080173 |
| Koala | 110207402 | 110206651 | 110192738 | 110218309 | 110192877 | 110192494 | 110215806 | 110206350 |
| Opossum | 100012502 | 100023065 | 100030485 | 100028926 | 100024569 | 100024569 | 100016618 | 100031592 |
| Large Flying fox | 105298891 | 105311247 | 105300535 | 105297143 | 105299435 | 105305832 | 105307511 | 105290707 |
| Black flying fox | 102893139 | 102885783 | 102883309 | 102894339 | 102886964 | 102887576 | 102882020 | 102897878 |
| Brandt's bat | n/a | 106726084 | 102254065 | 102263365 | 102256301 | 102253862 | 102248205 | 102241961 |
| Egyptian Fruit bat | 107504204 | 107510338 | 107497975 | 107516132 | 107513557 | 107520536 | 107520668 | 107512067 |
| Natal long-fingered bat | 107543384 | n/a | 107531672 | 107545236 | 107535231 | 107525555 | 107528961 | 107542574 |
| Nine-banded Armadillo | 101413319 | 101436605 | 101428716 | 101433349 | 101446468 | 101416819 | 101432315 | 101437378 |
| Chinchilla | 102019379 | 106149433 | 102023985 | 102012469 | 102012469 | 102005145 | 102007963 | 102007185 |
| Marmota | 107151690 | n/a | 107145366 | 107158610 | 107142656 | 107150918 | 107150629 | 107141944 |
| Squirrel | 101967779 | n/a | 101975369 | 101970499 | 101964841 | 101966670 | 101970320 | 101966565 |
| Rat | 24252 | 24253 | 25410 | 78965 | 307403 | 498951 | 25610 | 298518 |
| House mouse | 12606 | 12608 | 110794 | 12977 | 12978 | 76527 | 12985 | 12986 |
| Tarsier | n/a | n/a | 103270257 | 103269079 | 103263368 | 103265658 | 103271792 | 103262364 |
| Coquerel's Sifaka | 105812560 | 105818634 | 105814323 | 105825541 | 105821583 | 105807969 | 105824115 | 105808649 |
| Gray mouse Lemur | 105876979 | 105872281 | 105864646 | 105865049 | 105858431 | 105875426 | 105872091 | 105871730 |
| Small eared Galago | 100954648 | 100952496 | 100962438 | 100942777 | 100952454 | 100944728 | 100951748 | 100966247 |
| Common Marmoset | 103796367 | 100387105 | 100396210 | 100386071 | 100406047 | 100397966 | 100411779 | 100393434 |
| Black capped squirrel monkey | 104651063 | n/a | 101034696 | 101036115 | 101043573 | 101051147 | 101040223 | 101042318 |
| Nancy Ma's night monkey | 105714505 | 105727379 | 105727188 | 105727909 | 105715381 | 105706809 | 105717036 | 105708161 |
| Gorilla | 101129792 | 101125586 | 101135146 | 101131872 | 101151662 | 101135627 | 101145303 | 101128750 |
| Chimpanzee | 746673 | 469969 | 743538 | 457127 | 462187 | 454217 | 454641 | 456756 |
| Orangutan | 100442871 | 100444456 | 100442592 | 100452144 | 100452144 | 100454127 | 100442480 | 100433338 |
| Southern Pig-tailed macque | 105495586 | 105470688 | 105499610 | 105479257 | 105466874 | 105491293 | 105472231 | 105494725 |
| Crab eating macque | 102119204 | 102129932 | 102144336 | 102141631 | 102124214 | 102125350 | 102123087 | 102132219 |
| Rhesus macque | 717153 | 713191 | 713442 | 702532 | 711512 | 709034 | 698961 | 713485 |
| Human | 1050 | 1051 | 1053 | 1435 | 1436 | 146433 | 1440 | 1441 |
