# Supplementary material for: In-silico analysis of myeloid cells across the animal kingdom reveals neutrophil evolution by colony-stimulating factors
Source: eLife. 2020 Nov 25;9:e60214. doi: 10.7554/eLife.60214 (PMC7717901; doi:10.7554/eLife.60214)
Supplement: Supplementary file 5. [file elife-60214-supp5.docx]

|  | Protein accession number | | | | |
| --- | --- | --- | --- | --- | --- |
|  | CSF1 | IL34 | CSF1R | CSF3 | CSF3R |
| Coelacanth | n/a | XP_014347918.1 | XP_005988381.1 | ENSLACT00000017057.1 | XP_014354127.1 |
| Australian ghost shark | n/a | n/a | n/a | n/a | n/a |
| Whale shark | n/a | XP_020366056.1 | n/a | n/a | n/a |
| Lamprey | n/a | n/a | XP_032831918.1 | n/a | n/a |
| Hagfish | n/a | n/a | n/a | n/a | n/a |
| Tropical clawed frog | XP_031752726.1 | XP_012817098.1 | NP_001008181.1 | XP_031750736.1 | XP_002938787.3 |
| African clawed frog | XP_018103478.1 | XP_018116128.1 | XP_018110449.1 | XP_018090192.1 | NP_001086935.1 |
| High Himalaya frog | n/a | XP_018429647.1 | XP_018410447.1 | XP_018093459.1 | XP_018432060.1 |
| Two-lined Caecilians | XP_029428870.1 | XP_029464772.1 | XP_029439419.1 | XP_029428110.1 | XP_029427085.1 |
| Microcaecilia Unicolor | XP_030077183.1 | XP_030059460.1 | XP_030068873.1 | XP_030076333.1 | XP_030074347.1 |
| Three toed box turtle | XP_026514752.1 | XP_024067767.1 | XP_024055561.1 | XP_024060977.1 | XP_026512990.1 |
| Painted turtle | XP_005311609.1 | XP_008172997.1 | XP_005295871.1 | XP_005294193.1 | XP_023959983.1 |
| Green Sea turtle | EMP32291.1 | EMP26886.1 | XP_027689474.1 | XP_007062033.1 | XP_027678975.1 |
| Chinese Softshelled turtle | XP_025045117.1 | XP_014424401.1 | XP_006138292.1 | XP_006117480.1 | XP_025046029.1 |
| Burmese python | XP_025023512.1 | n/a | XP_007431230.1 | XP_025022516.1 | XP_025029950.1 |
| Mainland tiger snake | n/a | n/a | XP_026540897.1 | XP_026535458.1 | XP_026529456.1 |
| Eastern Brown snake | XP_026562960.1 | n/a | XP_026552383.1 | XP_026573376.1 | [XP_026558607.1](https://www.ncbi.nlm.nih.gov/sites/entrez?cmd=Search&db=protein&term=XP_026558607.1&dopt=GenBank) |
| Central bearded dragon | XP_020665646.1 | n/a | XP_020662460.1 | XP_020661538.1 | XP_020656259.1 |
| Gecko | XP_015262040.1 | n/a | XP_015273713.1 | XP_015279009.1 | XP_015266368.1 |
| Anole | XP_008107645.1 | n/a | XP_008103042.1 | XP_008111552.1 | XP_008120048.1 |
| Gharial | XP_019369416.1 | n/a | XP_019361698.1 | XP_019362586.1 | XP_019368096.1 |
| Australian saltwater crocodile | XP_019401815.1 | XP_019391134.1 | XP_019390760.1 | XP_019405873.1 | XP_019403436.1 |
| American Alligator | XP_019348061.1 | KYO37022.1 | XP_014453140.1 | XP_019338060.1 | XP_019345365.1 |
| Chinese Alligator | XP_014381918.2 | XP_025067592.1 | XP_014374244.1 | XP_025058688.1 | XP_025071644.1 |
| Emu | XP_025967824.1 | XP_025963134.1 | XP_025978411.1 | XP_025928730.1 | XP_025959467.1 |
| Ostrich | n/a | XP_009678081.1 | XP_009670341.1 | n/a | XP_009681327.1 |
| Kiwi | XP_025912480.1 | XP_025944387.1 | XP_025919230.1 | XP_025928730.1 | XP_025941806.1 |
| Mallard | XP_027299420.1 | XP_027322258.1 | XP_027324439.1 | n/a | XP_027299762.1 |
| Chicken | XP_015154359.1 | XP_003641940.1 | NP_001308446.1 | AAA48694.1 | XP_015153142.1 |
| Helmeted Guinea Fowl | XP_021232589.1 | XP_021264591.1 | XP_021265934.1 | XP_021233905.1 | XP_021231322.1 |
| Turkey | XP_010726285.2 | XP_003209716.3 | XP_003210460.1 | XP_003213980.2 | XP_010721736.1 |
| Platypus | XP_028924054.1 | XP_028931578.1 | XP_028907374.1 | XP_028930501.1 | XP_028936276.1 |
| Koala | XP_020856610.1 | XP_020819333.1 | XP_020819935.1 | XP_020853271.1 | XP_020839302.1 |
| Opossum | XP_016285121.1 | XP_007477581.1 | XP_007473921.1 | XP_007482348.1 | XP_007492877.1 |
| Large Flying fox | XP_011365935.2 | XP_023379404.1 | XP_011369464.1 | XP_011381345.1 | XP_023385201.1 |
| Black flying fox | XP_024907999.1 | XP_015443169.1 | XP_006906453.1 | XP_006924747.1 | XP_024897237.1 |
| Brandt's bat | XP_005860170.1 | XP_014392245.1 | XP_005870530.1 | XP_014391633.1 | XP_005868671.1 |
| Egyptian Fruit bat | XP_016011070.1 | XP_036087272.1 | XP_016005859.2 | XP_016020183.2 | XP_016002685.2 |
| Natal long-fingered bat | XP_016078761.1 | XP_016053229.1 | XP_016066088.1 | XP_016057699.1 | XP_016075421.1 |
| Nine-banded Armadillo | XP_004448989.1 | XP_023439356.1 | XP_004453792.1 | XP_004450127.1 | XP_023438504.1 |
| Chinchilla | XP_005388959.1 | XP_005403544.1 | XP_005380644.1 | XP_005394398.1 | XP_005395257.1 |
| Marmota | XP_015360574.1 | [XP_015351587.1](https://www.ncbi.nlm.nih.gov/sites/entrez?cmd=Search&db=protein&term=XP_015351587.1&dopt=GenBank) | XP_015341602.1 | XP_015351258.1 | XP_015340745.1 |
| Squirrel | XP_005338193.1 | XP_005318566.1 | XP_013213596.1 | XP_005321857.2 | XP_005318156.1 |
| Rat | AAH74007.1 | NP_001020937.1 | XP_008770370.1 | EDM05943.1 | XP_017448771.1 |
| House mouse | AAH66187.1 | XP_036010261.1 | NP_001032948.2 | P09920.1 | XP_006502775.1 |
| Tarsier | XP_008064843.1 | XP_008061504.1 | XP_008059241.1 | XP_008067483.1 | XP_008058174.1 |
| Coquerel's Sifaka | XP_012518836.1 | XP_012496963.1 | XP_012513771.1 | XP_012516942.1 | XP_012497813.1 |
| Gray mouse Lemur | XP_012607146.1 | XP_012627896.1 | XP_012597012.1 | XP_012621329.1 | XP_012620648.1 |
| Small eared Galago | XP_023365620.1 | XP_003791421.1 | XP_003782093.1 | XP_003786456.1 | XP_012659644.1 |
| Common Marmoset | XP_035108313.1 | XP_035138089.1 | XP_035144285.1 | XP_002763641.2 | XP_035106721.1 |
| Black capped squirrel monkey | XP_010342308.1 | XP_003939980.1 | XP_003934130.1 | XP_010328966.1 | XP_010346372.1 |
| Nancy Ma's night monkey | XP_012323507.1 | XP_012292826.1 | XP_021520605.1 | XP_012307412.1 | XP_012294770.2 |
| Gorilla | XP_018869616.2 | ENSGGOT00000013112.3 | XP_018882731.1 | XP_004041804.1 | XP_018871002.1 |
| Chimpanzee | XP_009428123.1 | [XP_511082.1](https://www.ncbi.nlm.nih.gov/sites/entrez?cmd=Search&db=protein&term=XP_511082.1&dopt=GenBank) | XP_024103186.1 | XP_009430519.1 | XP_024213682.1 |
| Orangutan | XP_024113270.1 | XP_002826665.1 | XP_024103186.1 | XP_009250119.1 | XP_024093579.1 |
| Southern Pig-tailed macaque | XP_011735483.1 | XP_011755838.1 | XP_011714318.1 | XP_011723578.2 | XP_011761757.1 |
| Crab eating macaque | XP_015287003.1 | XP_005592551.1 | XP_005558297.1 | XP_015293940.1 | XP_005544002.1 |
| Rhesus macaque | XP_028686403.1 | AFJ70629.1 | XP_001107711.4 | XP_001095097.2 | XP_014990976.1 |
| Human | P09603.2 | AAH29804.1 | AAH47521.1 | AAH33245.1 | AAN05790.1 |
